# Blood residence time to assess significance of coronary artery stenosis

**DOI:** 10.1038/s41598-020-68292-9

**Published:** 2020-07-15

**Authors:** Javad Hashemi, Shesh Rai, Shahab Ghafghazi, R. Eric Berson

**Affiliations:** 1 Department of Chemical Engineering, University of Louisville, Louisville, KY USA; 2 Department of Bioinformatics and Biostatistics, University of Louisville, Louisville, KY USA; 3 Department of Medicine, University of Louisville, Louisville, KY USA

**Keywords:** Interventional cardiology, Diagnosis

## Abstract

Coronary artery stenosis is a narrowing of coronary lumen space caused by an atherosclerotic lesion. Fractional flow reserve (FFR) is the gold standard metric to assess physiological significance of coronary stenosis, but requires an invasive procedure. Computational modeling in conjunction with patient-specific imaging demonstrates formation of regions of recirculatory flow distal to a stenosis, increasing mean blood residence time relative to uninhibited flow. A new computational parameter, mean blood residence time (Blood_RT_), was computed for 100 coronary artery segments for which FFR was known. A threshold for Blood_RT_ was determined to assess the physiological significance of a stenosis, analogous to diagnostic threshold for FFR. Model sensitivity and specificity of Blood_RT_ for diagnosis of hemodynamically significant coronary stenosis was 98% and 96% respectively, compared with FFR. When applied to clinical practice, this could potentially allow practicing cardiologists to accurately assess the severity of coronary stenosis without resorting to invasive techniques.

## Introduction

One million invasive coronary angiographies (ICAs) are performed on a yearly basis in the US and approximately half of these patients undergo percutaneous coronary intervention (PCI) and stent placement^1^. In patients for whom the indication for ICA is stable coronary artery disease (CAD) or chest pain, frequently, the interventional cardiologist encounters coronary stenosis of 40–90% and a decision has to be made with regards to revascularization (stent placement). The prevailing clinical practice is to visually estimate the degree of coronary blockages; patients with a stenosis ≥ 60–70% would then undergo PCI while others would receive medical therapy despite the fact that the totality of randomized trial data to date has not shown an improvement in patients’ outcomes when revascularization is guided by degree of stenosis alone^2,3^. Although, there is a fairly consistent relationship between the degree of coronary stenosis and coronary blood flow, the relation between the two is complex and there remains a disconnect between them in a significant number of cases^4^.

Accurate quantification of coronary stenosis is crucial in order to provide optimal medical care for patients. FFR, measured by placing a pressure wire across a stenotic lesion, is the gold standard method to determine the severity of coronary stenosis but requires an invasive medical procedure^5^. FFR is the ratio of maximal blood flow distal to a stenotic lesion to maximal flow in the same artery if hypothetically normal^6^. Normal FFR is 1 and an FFR ≤ 0.80 is considered hemodynamically significant^7^. The challenge is that measurement of FFR is an invasive extra step that is expensive, time consuming and associated with higher risk of complications, hence the slow acceptance rate of the technology by interventional cardiologists. Recent advances in computational fluid dynamics (CFD) enable calculation of coronary flow and pressure fields from anatomic image data^8^. Noninvasive calculation of FFR, or virtual FFR (vFFR), has been performed on images obtained from ICA or CT coronary angiography (CTCA)^9,10^. This method applies CFD to determine the physiologic significance of CAD without additional imaging, administration of medications, or use of pressure-wire and hyperemia.

Determining vFFR accurately depends on accuracy of the geometric renderings and model inputs. Empirical resistance boundary conditions at every coronary outlet are typically used^11–15^ but determining accurate values remains a dilemma^15^. Published data reports 6–12% combined false positive and false negatives for vFFR as compared to FFR^11,13,16^. Both FFR and vFFR are a function of pressure loss, a form of energy loss due to friction between fluid and the walls or between layers of the fluid itself. There are additional significant frictional losses around bends and through constrictions. In blood flow through stenotic arteries, recirculation regions are known to form distal to the stenosis^14,17–19^, which also present a major source of frictional and hence pressure loss. Blood is typically modeled as laminar, although localized regions of turbulence can exist in a recirculation region, and not accounting for the turbulent energy dissipation may reduce the accuracy of the predicted pressure loss. Even if modeled as turbulent, the velocity terms are still generally empirical.

Residence time is an indicator of flow trajectory and mixing. Deceleration of blood flow during the cardiac cycle yields large recirculation zones distal to stenosis leading to protracted path lengths and greater residence times. Kunov et al.^20^ showed experimentally, using tracer particles, that volumetric residence times were elevated in the separation zone distal to the stenosis. Cao et al.^18^ measured increasing residence time of tracers downstream of stenosis using a laser light sheet flow visualization method with pseudo-color display. Himburg et al.^21^ introduced relative residence time (RRT) to investigate the effect of the residence time of solutes and formed elements of the blood on atherosclerotic process, although RRT is indirectly computed from time-averaged wall shear stress (WSS) and oscillatory shear index (OSI) rather than directly from tracer measurements.

CFD can compute residence time in flow systems, which requires solving time dependent tracer concentrations after the velocity distribution is obtained. Computational time can be extensive even for steady flow, and time is critical if the technique is to be employed in real time in the cardiac catheterization lab. Mean age theory provides a computationally efficient method for computing residence time or “age” of fluid, where “age” refers to the amount of time a parcel of fluid resides between two boundaries. Sandberg^22^ derived the steady scalar transport equation for mean age based on the work of Spalding^23^, although limitations in computing power at the time made it impractical to put into practice. Further, conventional residence time theory is a measure of flow distribution at the exit of a continuous system whereas mean age theory provides spatial resolution throughout the interior of the flow domain. Since then, mean age has been demonstrated in a variety of applications for both single phase^24,25^ and multiphase flow^26,27^. Multiphase modeling would be beneficial if, for example, one wanted to determine the age of blood components such as cells, platelets, and plasma, etc. independent of each other.

We present here a new non-invasively determined metric, dimensionless Blood_RT_, to assess the physiological significance of coronary artery stenosis analogous to the coronary stenosis assessment based on FFR. The method to determine the metric is presented here and is based on CFD in conjunction with patient specific imaging to compute mean residence time of blood passing through stenotic coronary artery segments. We determined a threshold for Blood_RT_ and report the sensitivity and specificity of this metric to establish diagnostic accuracy compared with the gold standard pressure wire FFR threshold. We then tested our metric in one hundred coronary arteries with known pressure-wire FFR for clinical validation.

## Materials and methods

### Patient population

One hundred arteries from ninety patients who had undergone coronary angiography and FFR measurements for clinical indications in two affiliated hospitals in our institution were retrospectively included in this study. Patients’ characteristics are detailed in Table 1. Patients with stenosis in a major epicardial artery (left anterior descending artery [LAD], left circumflex [LCx]/obtuse marginal [OM] and right coronary artery [RCA]) were eligible for inclusion in the study. Exclusion criteria were: significant ostial left main or ostial RCA disease, coronary arteries with bifurcational lesions, and coronary arteries distally protected by bypass grafts. All lesions included in the study had documented adenosine administration and pressure-wire FFR recording, as well as suitable angiographic projections for Three-dimensional (3D) reconstruction. The Institutional Review Board at the University of Louisville approved the study protocol used for patient cases and all research was performed in accordance with relevant regulations. As the study was retrospective, and non-interventional in nature with minimal risk to subjects, the IRB granted waiver of informed consent.

**Table 1:** Clinical characteristics of patients.

Total patients	90
Total vessels	100
Age	63.3±27.6
Male gender	57 (63.3%)
Hypertension	87 (96.6%)
Diabetes mellitus	40 (44.4%)
Current smoker (last 1 year)	34 (37.7%)
History of prior myocardial infarction	35 (38.9%)
History of prior PCI	42 (46.6%)
History of prior CAD	57 (63.3%)
Hyperlipidemia	74 (82.2%)
family history	29 (32.2%)
Vessel disease	
Single-vessel	83 (83%)
Two-vessel	4 (4%)
Three-vessel	3 (3%)
total vessels	100

### 3D rendering

Three-dimensional (3D) reconstruction of coronary arteries was performed with the CAAS 7.5 QCA-3D system (Pie Medical Imaging, Maastricht, The Netherlands)^28^. In brief, two two-dimensional (2D) angiographic images encompassing the stenosis of interest, in images 30° apart, were used to generate a 3D rendering of the segment of interest, in the end-diastolic frames.

### CFD modeling

**Figure 1:**
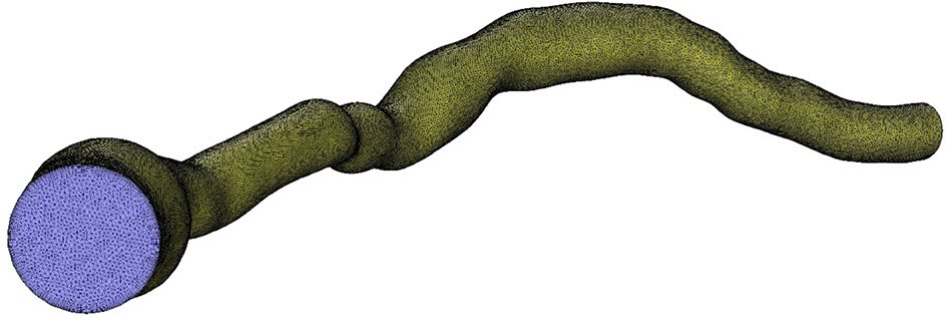
Sample meshed artery segment.

Blood flow in coronary arteries was simulated using ANSYS Fluent 17.0. Reynolds numbers were between 128–1501 in the region of stenosis, so flow was modeled as Laminar^14^. Blood density was 1045 kg m^−3^^29^. Blood viscosity was modeled as Newtonian. The value for each patient was determined from the patient’s percent contribution of plasma and red blood cells from lab measurement of the hematocrit. A viscosity of 0.008 kg m^−1^ s^−1^ was assumed for 100% hematocrit and 0.001 kg m^−1^ s^−1^ for 0% with a linear variation between these values^30^. The range of patients’ viscosities was 0.0035–0.0045 kg m^−1^ s^−1^ Anecdotally, a small change in viscosity value did not appreciably affect the residence time. Unstructured computational meshes (Fig. 1) were built as tetrahedral shaped cells using ANSYS Mesher 17.0. An optimal node count of 542,000 was determined by mesh sensitivity analysis of mean residence time for an artery with a volume of 4.04 ×10^−8^ m^3^, and then scaled accordingly for the size of the coronary segment for each case. A sensitivity analysis determined an optimal time step size of 0.01s.

**Figure 2:**
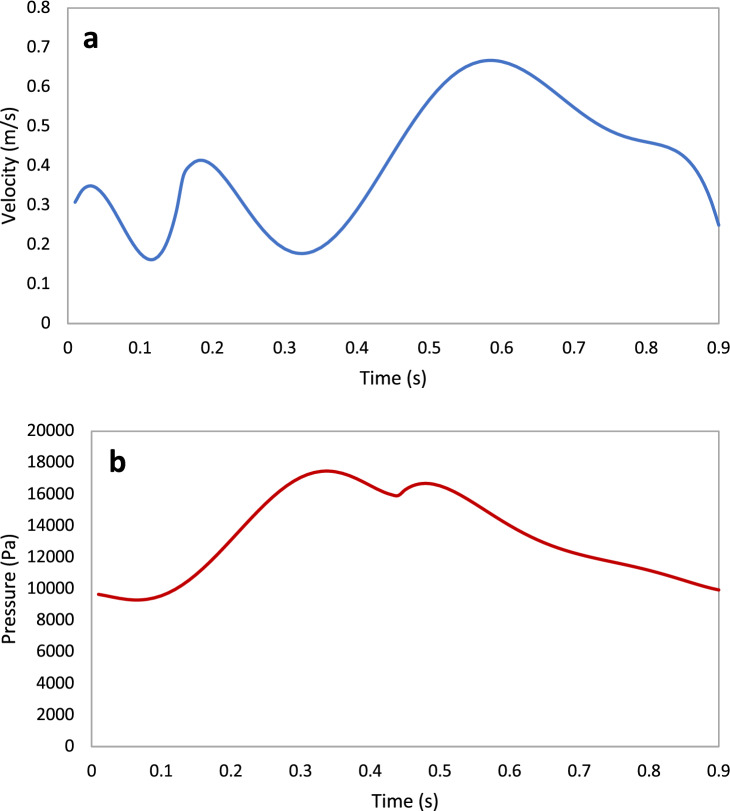
Sample hyperemic boundary conditions: (**a**) velocity inlet (**b**) pressure outlet.

The inlet boundary condition was a transient velocity waveform (Fig. 2a) representing the coronary blood cycle as previously published^31^ and entered into the ANSYS Fluent solver in a user defined function as a polynomial expression representing the waveform. The waveform was scaled for each patient by multiplying the coefficients of the polynomial by the ratio of an individual’s blood velocity to the velocity of the original waveform. The outlet boundary condition was a pressure waveform (Fig. 2b), and was similarly scaled by the pressure measured for each patient.

The goal of this work was to develop and validate a CFD based model for Blood_RT_. The challenge with inlet boundary conditions is well known ^16,28,32^. Therefore, we utilized a previously validated CFD model^11,33^ to calculate volume flow rate from clinically measured pressure-wire FFR. In detail, we initially calculated virtual FFR with the previously validated CFD model and varied volume flow rates until virtual FFR was within 1% of pressure wire measured FFR, concluding that the respective volume flow rate was accurate. This method was repeated for all patient cases reported in this study. A uniform velocity profile was assumed by dividing the volume flow rate by the inlet diameter of the segment.

Once the flow field was established, mean residence time was computed according to previously described CFD applications using “mean age theory”^25–27^. The general theory is briefly summarized here. Begin with the advective-diffusive equation:

1 $$\frac{\partial C}{\partial t}+\nabla\!\cdot\!\left(uC\right)=\nabla\!\cdot\!(D\nabla C)$$

where *C* is concentration, *t* is time, *u* is velocity and *D* is the diffusion constant. Mean age as a function of spatial distribution (x) is defined as:

2 $$a\left(x\right)=\frac{{\int }_{0}^{\infty }tC\left(x,t\right)dt}{{\int }_{0}^{\infty }C\left(x,t\right)dt}$$

Modifying (1) and incorporating (2) leads to:

3 $$\nabla\!\cdot\!(ua)=\nabla\!\cdot\!D\nabla a+1$$

This final equation provides the transport for mean age. Equation (3) is then solved secondary to the flow solution obtained by CFD to give a spatial distribution of the mean age scalar throughout the entire flow-field. Age typically refers to elapsed time of material at any location since entering the system. Residence time typically refers to elapsed time of material at the exit. Mean age at the exit, which is equivalent to mean residence time, was computed for each time increment and averaged over the duration of one complete pulse to yield the Computed Mean Residence Time in Equation (4).

### Computing hardware and compute time

Each case was run in parallel on 18 2.3 GHz 16 GB processors with a run time on the order of 12 hours. This covers the combined time for modeling the flow field and computing residence times.

### Statistical analysis to determine Blood_RT_ threshold

Observations were grouped into two groups, abnormal pressure-wire FFR (≤ 0.80) and normal pressure-wire FFR (> 0.80). Receiver operator characteristic (ROC) curve analysis was performed. Sensitivity and specificity of Blood_RT_ were calculated along with their 95% confidence intervals using logistic regression analysis to determine the optimal threshold for Blood_RT_. Except for the patient characteristics, analyses were performed on a per-vessel basis. A two-sided *p* value < 0.05 was considered significant. Blood_RT_ and FFR were compared using Bland-Altman analysis. The correlation between Blood_RT_ and FFR was studied using the Pearson (r) correlation coefficient.

## Results

### Pathlines

**Figure 3:**
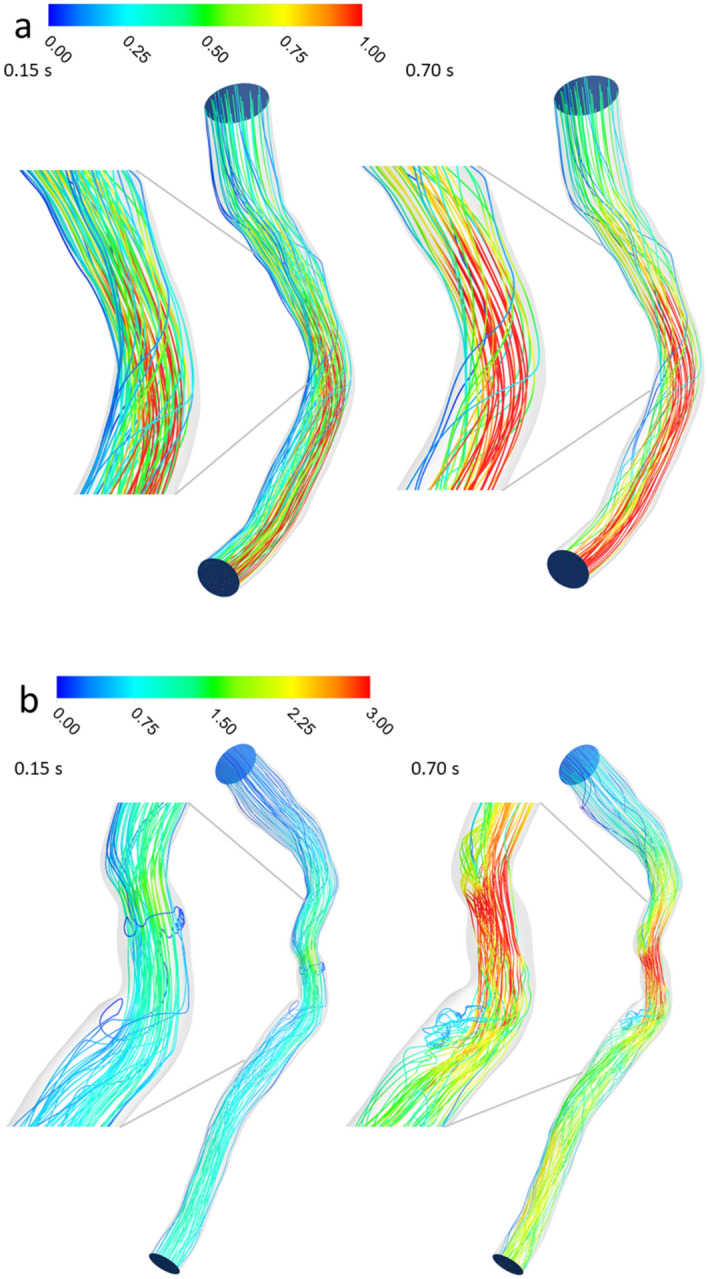
Blood flow pathlines (m/s) (**a**) for patient A and (**b**) for patient B during both systole (0.15s of pulse) and diastole (0.7s of pulse).

Blood flow pathlines are shown in two left anterior descending (LAD) artery segments as representative examples of one case above and one below the FFR threshold (Fig. 3a,b). Patient A had a non-significant stenosis with FFR equal to 0.94 and patient B had a significant stenosis with FFR equal to 0.63. Pathlines remain relatively ordered for Patient A during both systole (at 0.15s of the pulse) and diastole (at 0.70s of the pulse), while pathlines reveal a small but noticeable region of low velocity recirculation and holdup distal to the stenosis, especially during diastole (Fig. 3b). In Patient A, the maximum velocity during diastole was only about ~40% greater than the inlet velocity (~1.0 m/s compared to ~0.72 m/s) at this point in the pulse input (Figure 2a shows the velocity input pulse for Patient A), while for Patient B the maximum velocity was about 650% greater than the inlet velocity at this point (~3 m/s compared to ~0.4 m/s) in the pulse (pulse not shown).

### Wall shear stress

**Figure 4:**
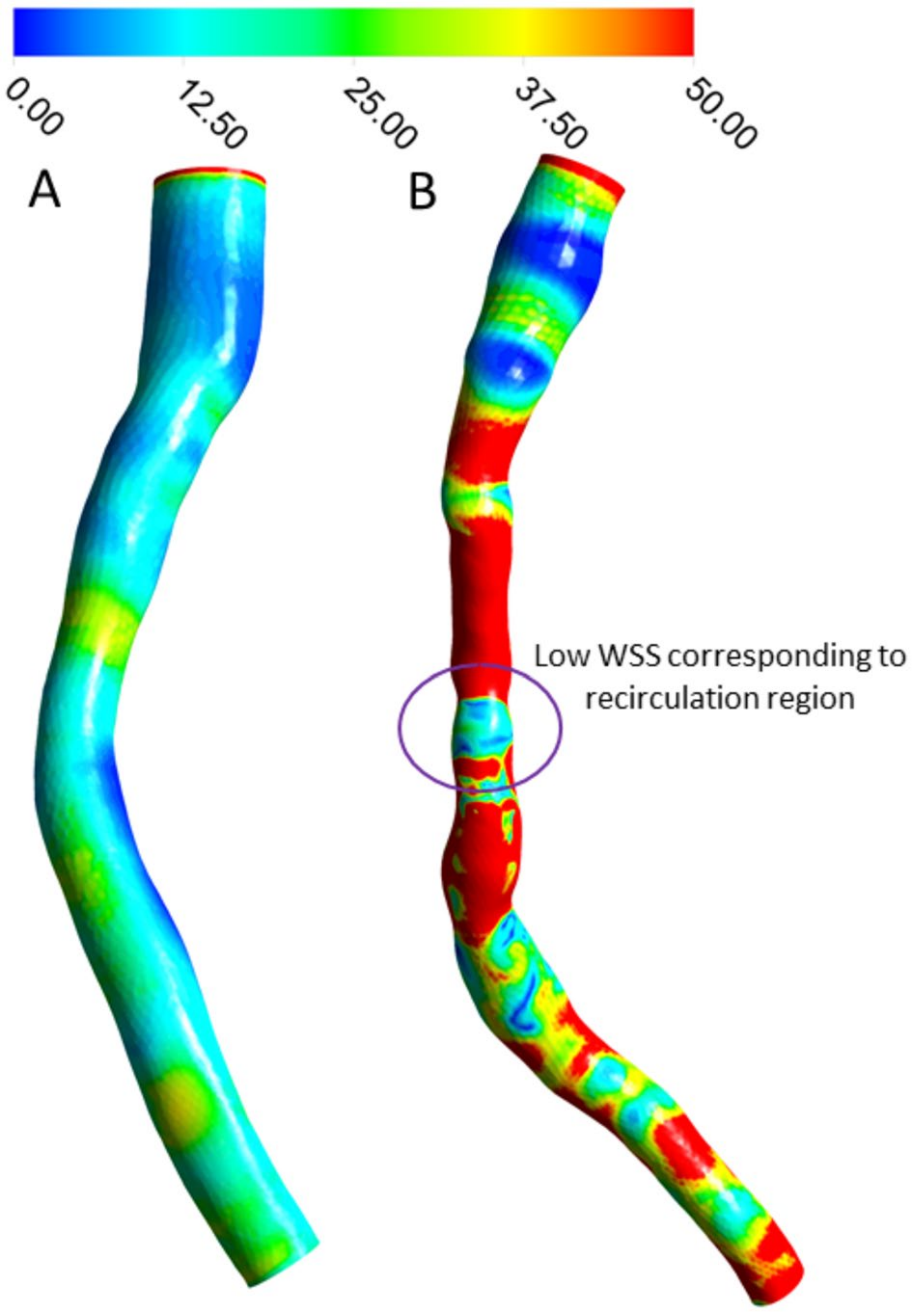
Wall shear stress (Pa) in two sample patients. A low WSS region is noticeable where recirculation occurs exiting the elongated stenosis of patient B.

Patient B has an elongated stenosis with high WSS throughout the stenosed region, but with a noticeable region of low WSS corresponding to the area of recirculation (Fig. 4). WSS is generally more ordered with little variability for Patient A. Both images are during systole, at 0.7s of the pulse.

### Mean residence time

**Figure 5:**
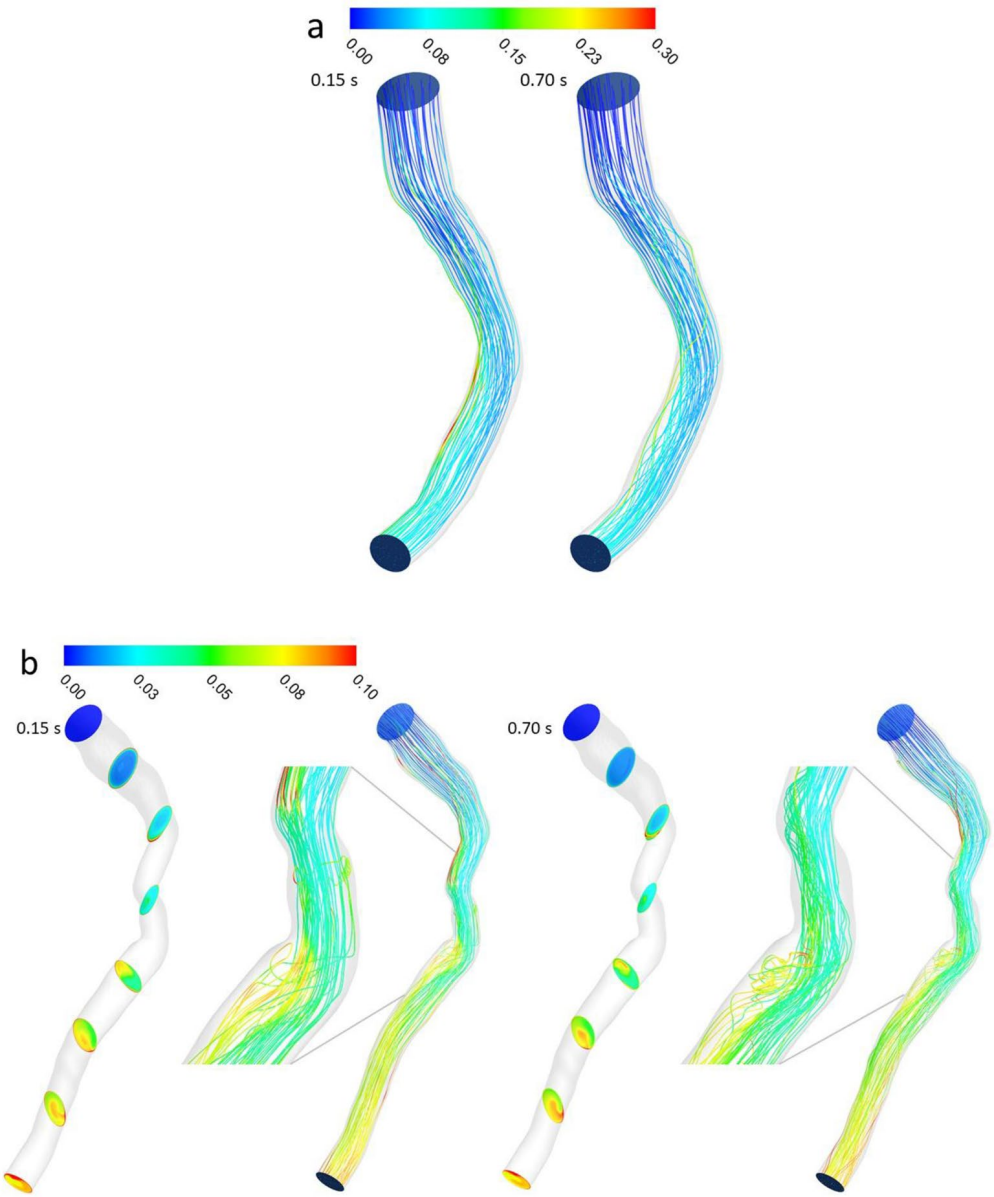
Mean residence time (s) pathlines (**a**) patient A and (**b**) patient B in systolic and diastolic phase.

Figure 5 shows pathlines colored by mean residence time for Patients A and B. The color indicates an increase in mean residence time from inlet to outlet for Patient A since there is no real holdup in this patient’s LAD segment. The overall mean residence time was 0.0817s, just 22% above its nominal mean residence time of 0.0670 s, where nominal mean residence time is defined as volume of the coronary segment divided by volume flow rate and represents the mean residence time that would be expected if flow was completely uninhibited. However, for Patient B, mean residence time in the recirculation region distal to stenosis is clearly high relative to the fluid passing in the main jet stream. The bulk of the mean time here is approximately 50% higher than the main jet stream, with certain points as much as three to four times higher. The overall mean time for this patient is 0.0796 s, while its nominal mean time was 0.0535 s, an increase of 49%.

**Figure 6:**
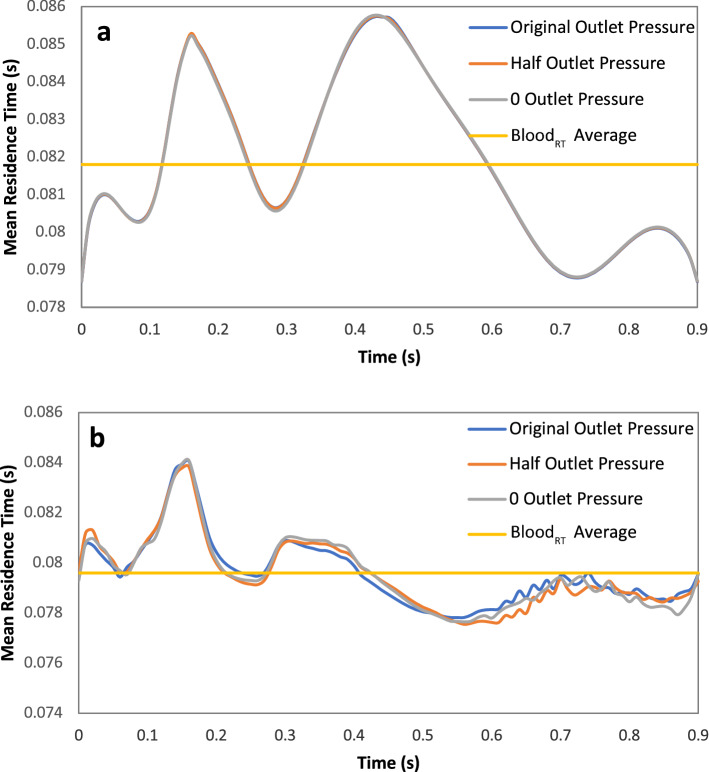
Mean residence time throughout one cardiac pulse for original outlet pressure, half the original outlet pressure, and 0 outlet pressure. (**a**) Patient A and (**b**) patient B.

Under normal conditions, mean residence time should increase during the systolic phase, when the velocity is generally lower, and decrease in the diastolic phase, when the velocity is generally higher. Mean residence time for patient A over the course of an entire pulse (Fig. 6a) reflects this, where the amplitudes are low when velocity amplitudes are high and vice-versa. Also, as the slope of the velocity increases, the slope of mean time decreases and vice-versa. Mean time adheres to this during systole for Patient B, but more or less levels off during diastole when it should be decreasing (Fig. 6b), which is reflective of the recirculation and holdup during the diastolic phase. The time in Figures 6a,b represents the mean exit residence time of blood that entered the arterial segment at a given time during the cardiac cycle, and the overall mean value is reported as the average of mean residence times over one complete cycle.

The pressure outlet boundary condition did not affect mean residence time for patient A (Fig. 6a) or Patient B (Fig. 6b)_._ Figure 6a shows mean residence time throughout one pulse for the original pressure, half the original pressure, and zero (gauge) pressure for patient A. Mean residence time for these three examples were 0.0818±0.00001s. Figure 6b shows the same for patient B with a mean residence time of 0.0796±0.00009 s.

### Blood_RT_ and threshold

Blood_RT_ is defined as a dimensionless parameter to account for varying length and volume of each arterial segment plus varying blood flow rates:

4 $${Blood}_{RT}=\left(\frac{ Nominal Mean Residence Time (s)}{Computed Mean Residence Time\left(s\right)}\right)$$

Nominal mean residence time is the volume of the segment divided by the flow rate, and represents the expected residence time in the absence of an obstruction or other feature causing disturbed flow. Computed mean residence time is the CFD derived result described in the Methods. Blood_RT_ should equal 1.0 for completely unobstructed and ordered flow, and will be a fraction of 1.0 when obstructed flow increases the residence time relative to the nominal mean time. The method for determining the computed mean residence time was validated by comparing the CFD computed time with the nominal residence time in a normal artery with 0% stenosis, in which case the two should be equal. In one such example, the computed mean residence time was 0.128 s and the nominal mean residence time was 0.126 s, yielding a Blood_RT_ = 0.98.

**Figure 7:**
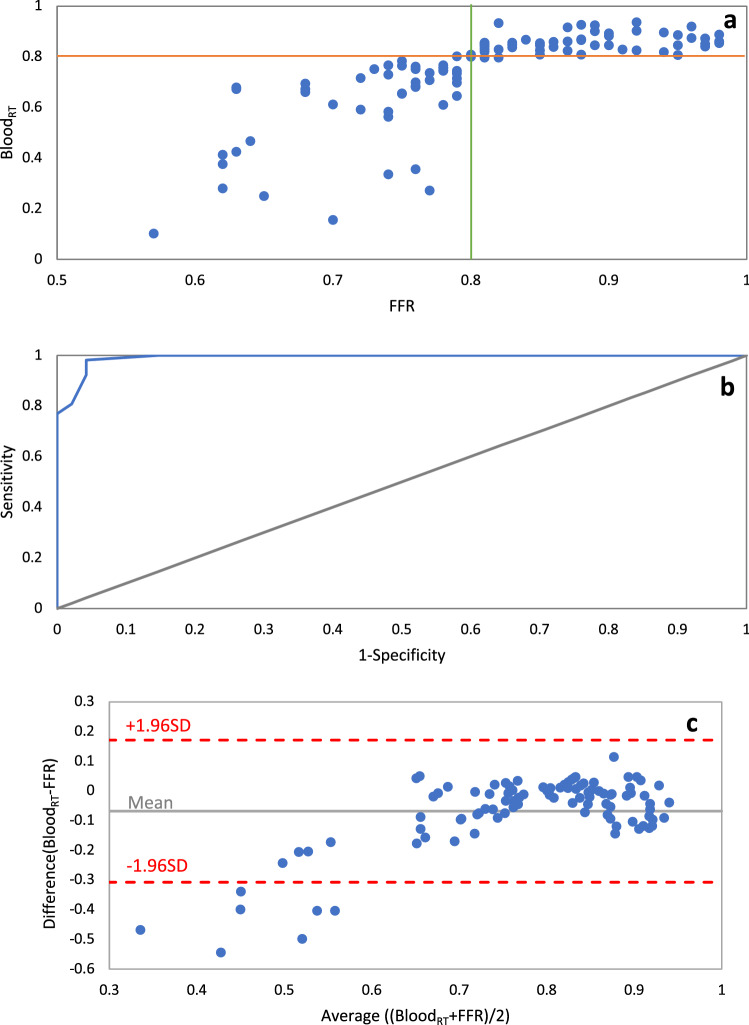
(**a**) Blood_RT_ and invasive FFR for 100 patient coronary arteries and (**b**) receiver operator characteristic (ROC) curve analysis for Blood_RT_ against the fractional flow reserve (area under curve=0.9959) (**c**) Bland–Altman analysis.

Mean residence time was first determined in 100 coronary arteries for which the gold standard pressure-wire FFR was known. Abnormal (FFR≤0.80) and normal (FFR>0.80) groups based on the pressure-wire FFR threshold are highly associated with a Blood_RT_ threshold of 0.80. There were 46 true negatives (46%), 51 true positives (51%), 1 false negative (1%) and, 2 false positives (2%) (Fig. 7a). The sensitivity and specificity (along their 95% confidence intervals) are 98% (88–100) and 96% (86–100) respectively, indicating strong ability for Blood_RT_ to predict whether FFR is above or below 0.80. These AUC, sensitivity, and specificity values compared favorably to various forms of virtual FFR (Table 2). The ROC curve is presented in Figure 7b. While the main objective was to determine the Blood_RT_ threshold for diagnostic accuracy, there was also a strong correlation between pressure-wire FFR and Blood_RT_ values (r=0.75, *p*< 0.001). Figure 7c presents a Bland-Altman plot for Blood_RT_ and pressure-wire FFR. The difference between pairwise Blood_RT_ and pressure-wire FFR is plotted against the mean of the two measurements and is suitable for investigation of any possible relationship between measurement error and the true value. Bland-Altman analysis indicates excellent agreement between Blood_RT_ and pressure-wire FFR.

**Table 2:** Statistical analysis comparison between Blood_RT_ and various forms of virtual FFR

Metrics	Case numbers	AUC	Sensitivity	Specificity
Blood_RT_	100	0.996	96	98
FFR_angio_^32^	184	0.97	88	95
QFR^10^	87	0.91	78	89
FFR_QCA_^16^	77	0.93	78	93
vFAI^28^	139	0.92	90.4	86.2
Virtual FFR-VIRTU-1^33^	35		71	100
Stenosis flow reserve (SFR)^34^	110		93	85

## Discussion

Pressure-wire FFR, the gold standard for diagnosing the physiological significance of coronary stenosis, is a function of pressure loss across the stenotic segment^34^. The altered, disordered flow due to stenosis leads to frictional loss between layers of fluid, fluid and the wall, and especially around bends, through constrictions, and in observed recirculation regions, resulting in loss of pressure^35^. Rather than measuring or computing pressure loss to quantify physiological significance, we have presented a new approach which directly quantifies altered flow trajectories through residence time, a well-established mixing metric that quantifies variations in time spent in a given volume due to variations in paths through the same volume.

While mean age theory has primarily been demonstrated in industrial systems^22,25,26,36^, it can also be useful for characterizing flow within vessels and human organs or their model counterparts. In this study, we employed mean age theory to characterize blood flow characteristics in coronary segments. Parameters such as relative velocity and WSS are indicative of changes in flow characteristics, but by themselves do not necessarily correlate to physiologic significance in stenotic coronary arteries^14^. On the other hand, mean residence time, especially relative to unobstructed flow, is a widely used established indicator of variance in flow. Two objects with equal volume and flow may have vastly different flow characteristics and, hence, mean residence time if their geometries or, in this case, anatomies differ.

Stenotic flows, which have been well characterized in several studies, exhibit flow separation downstream of the stenosis characterized by a central jet stream and secondary flow near the wall, with a strong shear layer in between^37,38^. The deceleration of flow during diastole is responsible for the conditions that create the secondary flow reversal downstream of the stenosis. The flow separation depends on the upstream flow velocity and diameter of the stenosis^39^. The velocity gradient and shear layer at the interface provide the potential for reversed flow due to the tangential force^40^. This effect occurred here just past the region of stenosis (Fig. 3b).

Mean residence time increased relative to nominal mean time due to flow characteristics distal to the stenosis zone, with practically no effect on residence time proximal to the stenosis. Even a small fraction of blood recirculating in the secondary flow region will cause the overall residence time to increase above the nominal value. Higher mean time in the recirculation region associated with Patient B was approximately 1.5–4× the surrounding fluid that passes uninhibited, contributing to the overall increase in mean time at the exit or, by definition, decrease in the dimensionless Blood_RT_. Blood_RT_ for patient B’s LAD with FFR=0.63, was 0.67. Both values indicate an extreme departure from their respective thresholds and are representative of severely disturbed flow due to an elongated stenosis.

While the recirculation pattern generally exists indefinitely, fluid that enters this region eventually crosses back into the primary flow stream at the boundary between the primary and secondary streams. Else, even with a small amount of indefinite fluid holdup, the mean residence time would approach infinity. The holdup time and variance from nominal residence time depends on the combination and interactions of factors such as velocity through the stenosed area, the size of the stenosis, and shape of the artery segment such as if it were a straight or bent segment.

The threshold between a hemodynamically significant or non-significant stenosis was also determined for our new Blood_RT_ metric. Blood_RT_ agreed with pressure-wire FFR in all but three cases on the hemodynamic significance of the stenosis. It is noteworthy that the few cases not in agreement were within ~0.5% of the statistically determined threshold; the Blood_RT_ of the two false positives were 0.796 and 0.797, and the Blood_RT_ of the false negative was 0.802. Both the Blood_RT_ and pressure-wire FFR thresholds equal to a dimensionless value of ~0.80. Blood_RT_ is a measure of relative time while pressure-wire FFR is a measure of relative pressure. The two are indirectly related through fluid flow phenomena, but it is only coincidental that they are equal. It is possible that the Blood_RT_ threshold shifts if more cases are added to the study, but given the strong statistical correlation, any shift would likely be minimal. The similarity in thresholds does not imply that values should be equal for individual cases as Blood_RT_ is a measure of time while FFR is a measure of pressure, however there was a close correlation and agreement between Blood_RT_ and pressure-wire FFR (r=0.753, *p*<0.0001). Patient B provides a sound example with FFR=0.63 and Blood_RT_ = 0.67.

There was a region of decreased WSS (Fig. 4) coinciding with the region of recirculation, which also coincided with increased mean residence time (Fig. 5). Previously, Himburg et al.^21^ introduced the concept of RRT, which is calculated from WSS and OSI rather than tracer measurements. Guerciotti et al.^41^ also reported an inverse relation between WSS and RRT due to disturbed flow. However, they also stated that RRT provides no direct information on the actual residence time of blood in a given region. While use of the “relative residence time” is in some way an indicator of disturbed flow, it is actually a misnomer as it is reported with units of inverse pressure. The dimensionless parameter Blood_RT_, on the other hand, is a true direct measure of residence time.

## Limitations

In this study, we used blood velocity as the inlet boundary condition. Although direct measurement of coronary flow velocities has been feasible for decades, it is an invasive and time consuming process. Papafaklis et al.^28^ used a predetermined flow rate for baseline and hyperemic conditions based on the literature. Others have used angiographic contrast flow rate to determine inlet velocity/volume flow rate^42^. For the purpose of this work, we calculated inlet volume flow rate from pressure-wire determined FFR through a previously validated CFD model^43^. This method was necessary in order to develop and validate the CFD based model for calculation of Blood_RT_ and future work will address the best strategy to determine inlet volume flow rate. For example, regression and machine learning type methods can be used to estimate volume flow rate from anatomical imaging and other readily available patient data. Regression and machine learning approaches are negligible, with regards to solution time, compared to the CFD computational time. Initial attempts using one such method suggests a variance of about 30% in flow rate will not affect overall Blood_RT_ values since both the expected nominal time and the actual computed time in the definition of Blood_RT_ both depend on the incoming flow rate^44^. Future studies will be needed to investigate the discrimination power of the Blood_RT_ model in quantification of coronary stenosis with the above techniques used for volume flow rate determination. Total computational time needs to be on the order of minutes to be clinically applicable. Future development will focus on scalable and GPU computing. Novel modeling techniques also show promise for achieving results within the desired time frame^45^.

## Conclusions

We developed a new computational based metric to assess the physiological significance of coronary stenosis without the use of invasive pressure-wire measurement. The dimensionless metric, Blood_RT_, is representative of the average time it takes blood to pass through a given arterial segment, and is indicative of the increase in time due to stenosis as compared to the nominal time for blood transit through that segment. Increase in mean residence time is due to a small region of recirculatory flow distal to stenosis as elucidated by model derived pathlines. The method was then applied to one hundred coronary arteries from patients who had already undergone pressure-wire FFR measurement for clinical indications. A threshold for Blood_RT_ was determined that demonstrates excellent discrimination in detecting hemodynamically significant from non-significant stenosis compared to the gold standard pressure-wire FFR.

### Ethical approval

The Institutional Review Board at the University of Louisville approved the study; IRB number 16.0378.
